# Effective endoscopy reporting in inflammatory bowel disease: a trainee’s guide

**DOI:** 10.1093/crocol/otag082

**Published:** 2026-07-17

**Authors:** Kofi Clarke, Matthew D Coates

**Affiliations:** Division of Gastroenterology and Hepatology, Department of Medicine, Penn State Health , 500 University Drive, Hershey, PA 17033, United States; Division of Gastroenterology and Hepatology, Department of Medicine, Penn State Health , 500 University Drive, Hershey, PA 17033, United States

## Introduction

Endoscopy reports are an integral part of inflammatory bowel disease (IBD) patient care. They provide critical information about activity and severity of disease, help assess response to treatment and facilitate surveillance for colon cancer and its precursors. In addition, endoscopic procedures play an important role in addressing specific questions from referring providers, including evaluation of concerning lesions, intervention in areas of disease-related strictures, and determining post-operative risk of disease recurrence.

Endoscopy charting software is utilized to provide a defined structure and format for reports, provide consistency as well as to incorporate validated scoring systems for IBD endoscopic findings when appropriate. The data captured, organized, and reported by the software also provides important information to support research.

Due to ongoing growing pressure impacting physician time and efficiency, there is a risk of creating reports that may not contain adequate detail to answer specific questions or provide appropriate guidance for clinical decision making to optimally manage patients with IBD. As such, it is critical that trainees learn how to write a report that effectively describes significant IBD-associated endoscopic findings, and relays the proper recommendations based on these results early in their training.

We have developed the following outline to provide guidance on key aspects of endoscopy reporting in patients with IBD. This paper includes recommendations regarding the use of validated IBD-associated scoring systems and explains how to appropriately respond to questions related to the endoscopy request.

## Considerations before writing the IBD endoscopy report

The first critical step in writing a good IBD-focused endoscopy report is to clarify key details related to the patient’s personal and endoscopic history, and to determine the primary goals of the procedure(s) being performed. In order to do this, the following questions should be answered:

1. What is the indication or clinical question being asked pertaining to IBD?
2. When was the last endoscopy done, and what were the findings related to IBD?
3. *What is the medication history and current medication?*
4. Is there a history of bowel-related surgery (related to IBD or otherwise)? If yes, what kind?
5. Will the procedure require biopsies, or other interventions such as dilation of strictures or endoscopic mucosal resection (EMR)?
6. Is there a mass or other lesion that requires follow-up? If so, are there specialized surveillance modalities (eg NBI, chromoendoscopy, etc.) that may be appropriate to employ?

## Obtaining consent

The process of informed consent cannot be over-emphasized. As described above, it is essential for the endoscopist to review all of the salient points related to the patient’s history, and the procedure(s) they are presenting for, before moving forward. The endoscopist should also review the key elements of the medical history and the purpose of the procedure(s) with the patient, using simple, uncomplicated language to verify appropriate understanding. In patients with IBD, the discussion should include an added focus on the risk of perforation related to potential dilation, lesion resection, and/or navigating surgical anastomoses when appropriate. When undertaking multiple procedures (eg EGD with colonoscopy or ileostomy) completion of separate consents is required after an appropriate discussion of the related risks, benefits, and alternatives for each procedure.

## Tracking your findings during the procedure

If possible, the endoscopist should utilize other staff in the room to document findings instead of committing every finding to memory. One potential approach is to succinctly but carefully describe key findings to be written down by a designated staff member. In such cases, it is important to routinely use closed-loop communication to ensure accuracy of the recorded information. For trainees in particular, having such a back-up system can prove invaluable for accurate documentation and reporting.

## Photodocumentation

As always, it is important to provide evidence that key landmarks, including the appendiceal orifice, Ileocecal valve and retroflexed, and *en face* views of the rectum, were adequately visualized. Additionally, all IBD-related findings should be captured.^1^ This includes demonstration of the severity and extent of inflammation (utilizing standardized scales such as the Mayo endoscopy sub-score or simple endoscopy score for CD) and any relevant complications, including strictures, masses, and fistulae. Finally, surgical interventions such as bowel-bowel anastomoses (including type, location, appearance, and state of inflammation), *other abnormal areas including areas suspicious for dysplasia*, and seton placements should be documented when encountered. *We also recommend the use of tattoos as clinically indicated to help locate suspicious areas at follow up procedures*. Additional documentation of any IBD-related findings during the perianal examination is essential.

## Writing the report

In order to efficiently describe and organize the information above, the report should be consistently structured, utilizing pre-set endoscopic software formatting and drop-down phrasing whenever possible.^2^ However, it is reasonable to manually add relevant descriptions when the former is inadequate. The following points should all be addressed as clinically appropriate:

1. Start the report with an expanded description of the indication IF the drop-down menu in the charting software is not adequate. *Additional details that are key to a thorough report and follow up care include patient’s disease type, medications, and exam indication.*
2. All disease-related findings should be described in terms of location and severity, using standardized scoring systems (eg Mayo Endoscopic Sub-score; SES-CD; Rutgeert’s Score after ileocolectomy).
3. Surgical interventions, including the type, location and appearance of anastomoses and ability to traverse these areas, should also be described.
4. Recommendations on appropriate clinical and endoscopic follow-up should be clearly documented.

Once the written portion of the report is completed, annotate the appropriate documents, including relevant descriptive phrases, and highlighting key findings on the images themselves as appropriate.

*In addition, we recommend examination with virtual or dye-based chromoendoscopy in high-risk patients if there is no previous documented examination following concerns about findings on high-definition white-light examination following. These include patients with a history of Primary Sclerosing Cholangitis, extensive disease duration, and previous documented dysplastic lesions -either visible or noted from non-targeted biopsies. Other patients in this group are patients with non*–*IBD-related increased risk of colon cancer eg relevant family history of colon cancer or genetic syndromes with increased risk of colon cancer.*

## Refining the reports

Discuss with your faculty, and seek feedback regularly to evaluate your progress. You can also periodically assess your own progress. *Consider incorporating details used by experienced IBD faculty in prior reports to enhance the quality of your reports*. Quality metrics on bowel preparation, including withdrawal time and total procedure time, are captured by the reporting software. It is helpful to intermittently evaluate your progress by reviewing your metrics at regular intervals.

Key points summary box

1. Prepare for endoscopic intervention by learning each patient’s history and clarifying the goals of each planned procedure
2. Obtain consent by carefully explaining the goals, risks, and technical aspects of each procedure and ensure patients understand these concepts
3. Note key elements during each procedure
4. Photodocument key landmarks and findings during each procedure
5. Write a standardize report that includes (1) appropriate description of the indication, (2) description of the disease status, extent, and complications, (3) prior surgical history (as necessary), and (4) subsequent recommendations
6. Refine your approach and seek feedback from supervising faculty

## Funding

None declared.

## Conflicts of interest

The authors have no relevant conflict of interest or funding sources to report.

## Data Availability

Not applicable.
